# SUMOylation machinery protein, PIAS4 role in breast cancer cell proliferation and drug sensitivity

**DOI:** 10.1007/s11033-025-11423-0

**Published:** 2026-01-30

**Authors:** Mohammed A. M. Salih, Mohamed M. A. E. L. Salem, Muhammad Ali Shahid, Hussein A. S. Elrewey, Ritchie Williamson, Sriharsha Kantamneni

**Affiliations:** 1 Institute of Cancer Therapeutics, University of Bradford, Bradford, UK; 2 School of Pharmacy, Optometry and Medical Sciences, Faculty of Health and Social Care, University of Bradford, Bradford, UK; 3 University of Bradford International College, University of Bradford, Bradford, UK; 4 Present Address: Department of Health and Social Care, Scholars School System, Leeds Trinity University, Leeds, UK; 5 Faculty of Pharmacy, Tobruk University, Tobruk, Libya

**Keywords:** Breast cancer, MCF-7, Naked mole-rat, PIAS4, SUMOylation

## Abstract

**Purpose:**

Breast cancer is a significant global health issue, with resistance to doxorubicin (DOX) posing a major challenge to effective treatment. SUMOylation, a post-translational modification process, is linked to cancer progression and therapy resistance. PIAS4, a SUMO E3 ligase involved in maintaining genome stability and stress response, may play a role in these mechanisms. However, its function in breast cancer progression and DOX resistance remains uncertain. This study investigates the potential role of PIAS4 in mediating DOX resistance in breast cancer.

**Methods and results:**

Naked mole-rats (NMRs) are cancer-resistant rodents with improved genome maintenance, yet the role of SUMOylation in this trait remains unclear. SUMOylation machinery gene expression levels are investigated using qPCR in NMR tissue in comparison with carcinogenic breast cancer (MCF-7) cell line. Functional studies are performed in MCF-7 cells overexpressing PIAS4 to demonstrate effects on proliferation, invasion, drug sensitivity, and protein expression in the presence and absence of DOX treatment. While most SUMOylation genes were expressed at low levels in NMR intestinal tissues, PIAS4 showed higher expression compared to MCF-7 cells. PIAS4 overexpression in MCF-7 cells significantly decreases colony formation, invasiveness, and resistance to DOX. Western blot analysis showed downregulated Bcl-2 protein levels after DOX treatment, indicating a potential role in apoptosis evasion.

**Conclusion:**

PIAS4 expression level plays a role in breast cancer cell survival, invasiveness, and chemoresistance, partly by altering anti-apoptotic pathways. These findings position PIAS4 as a potential biomarker and therapeutic target for overcoming resistance to anthracycline-based therapies in breast cancer.

**Supplementary Information:**

The online version contains supplementary material available at 10.1007/s11033-025-11423-0.

## Introduction

Every year, just over 2.3 million new breast cancer (BC) cases are diagnosed in women [1]. Early detection and treatments, such as anthracyclines (e.g., doxorubicin), have increased survival rates, although treatment resistance and cancer recurrence remain significant challenges [2]. Doxorubicin (DOX) works by intercalating with DNA, inhibiting topoisomerase II, and generating reactive oxygen species (ROS) to induce cell death [3]. However, cancer cells can develop resistance to DOX through increased drug efflux, changes in topoisomerase II (reliance on beta-isoform), and modulation of cell survival pathways [4]. Previous studies indicate that SUMOylation plays a role in these adaptive responses. PIAS4, a component of this process, helps oncoproteins persist longer and facilitates DNA double-strand break repair [5, 6].

E3 SUMO (Small Ubiquitin-like Modifier) ligases belong to the Protein Inhibitor of Activated STAT (PIAS) family. These ligases have an influence on post-translational modification, mainly SUMOylation and change protein stability, subcellular localisation, and transcriptional activity [7]. PIAS4 (also called PIASγ) is one of four human PIAS proteins (PIAS1-4). They play a crucial role to repress transcription, remodelling chromatin, and maintain genome stability [8, 9]. PIAS4 dysregulation has been linked to several cancers however, how PIAS4 helps cancer growth and resist treatment remains unclear [10].

The naked mole-rat (NMR) is a one-of-a-kind rodent model that lives an exceptionally long life (more than 30 years) and resists cancer with improved genome stability and DNA repair systems [11]. By comparing expression levels of genes/proteins between NMRs, human cells and/or rodents, there is potential to gain useful insights such as molecular changes that support resistance to tumour growth [12, 13]. NMR intestinal tissue has a high proliferative capacity, provides a relevant non-cancerous model for assessing baseline expression of genes involved in SUMOylation. Comparing NMR tissue with human breast cancer cell lines enables the identification of alterations in SUMOylation components that may contribute to tumorigenesis.

In this study, we determined SUMOylation pathway mRNA expression profiles. These include SUMO1/2/3, SENP1/2/3/5/6/7, SAE1, Uba2, Ubc9, and PIAS1-4. We compared their expression between NMR intestinal tissues and human breast cell lines. We found higher expression of PIAS4 in NMR tissue compared to breast cells. The role of SUMOylation in NMRs remains largely unexplored; this study provides novel data and the first analysis of the SUMO pathway gene expression in NMR tissue.

We then recapitulated the expression of PIAS4 in human breast cancer cell line MCF-7 using over-expression constructs as a proof of concept. Our results show that increased PIAS4 protein expression leads to a decrease in IC50 of DOX, reduction in colony formation and invasion. Bcl-2 was selected for analysis based on evidence linking PIAS4 activity to the regulation of anti-apoptotic signalling, chemoresistance, modulation of transcription factors that influence Bcl-2 expression [14]. Our data showed a decrease in Bcl-2 levels following PIAS4 overexpression, particularly under DOX treatment. Although more work is needed in multiple cell lines, the present findings suggest that PIAS4 has a role in cancer cell proliferation and survival, which might serve as a potential target for therapy in breast cancer.

## Materials and methods

### Primer sequences design

SUMOylation primer sequences were designed for both NMR and Human using two bioinformatics tools: NCBI Primer-BLAST and PrimerQuest™ (Integrated DNA Technologies, www.idtdna.com/PrimerQuest/). Primer pairs were selected based on optimal melting temperature (Tm), GC content (40–60%), and minimal secondary structure or dimer formation. Housekeeping gene β2-microglobulin (B2M) was used as a reference gene for normalisation of expression data. All primers were synthesised by Sigma-Aldrich (UK) at standard desalted purity and validated by conventional PCR and melt curve analysis in qRT-PCR to confirm specificity and single product amplification. See supplementary Table 1 for primer sequences.

### Pairwise sequence alignment

Basic Local Alignment Search Tool (BLAST) https://blast.ncbi.nlm.nih.gov/Blast.cgi. was used to identify similar regions of human and NMR SUMOylation mRNA and protein sequences. Sequences were selected from NCBI FASTA and both sequences of accession numbers and in the FASTA format were entered in BLAST. Algorithm parameters of the matrix option were selected and the query and subject from the sequences were gathered for scoring. See supplementary Table 2 for pairwise sequences alignment.

### Cell lines and culture conditions

Non-malignant breast epithelial cells (MCF-10 A) at passage 3 (P3) and human breast cancer cells (MCF-7) at passage 6 (P6) were provided by the Institute of Cancer Therapeutics (ICT) at the University of Bradford (UoB) originally sourced from ATCC. Both cells were cultured in RPMI 1640 medium supplemented with 10% fetal bovine serum (FBS) (Sigma-Aldrich, United Kingdom), 10 ml L-Glutamine (Gibco, United Kingdom), and 1.2% (v/v) penicillin/streptomycin (ThermoFisher Scientific). All cells were maintained at 37 °C with 5% CO2.

### Quantitative real-time PCR (qPCR)

Total RNA was extracted from MCF-10 A, MCF-7, and NMR tissues using the RNeasy Mini Kit (Qiagen, UK). cDNA synthesis was performed using 1 µg RNA and the High-Capacity cDNA Reverse Transcription Kit (Applied Biosystems). qPCR was carried out using SYBR™ Green Master Mix on a QuantStudio™ 5 Real-Time PCR System (Applied Biosystems). Reactions were run in triplicate (95 °C for 10 min; 40 cycles of 95 °C for 15 s and 60 °C for 1 min). Primers were designed for SUMO pathway genes (*SUMO1/2/3*,* PIAS1-4*,* SENP1–7*,* SAE1*,* UBA2*, and *UBE21*). *B2M* was used as the endogenous control. Relative gene expression was calculated using the ΔCt method and melt curve analysis confirmed amplification specificity.

### Expression plasmid design and extraction

Expression of dTomato (pRP[Exp]EGFP/PuroCMV > dTomato) and PIAS4 (Myc/hPIAS4[NM_015897.4]) plasmids was designed using the VectorBuilder online tool (www.vectorbuilder.com). The GenElute Plasmid Midiprep Kit (Sigma Aldrich, United Kingdom) was used to extract the plasmid DNA according to the manufacturer’s instructions.

### Plasmid transfection and stable cell lines

MCF7 breast cancer cells (3 × 10⁵ cells/ml) were seeded in a 6-well plate and incubated at 37 °C for 24 h. After replacing the medium with serum-free medium, plasmid solutions of Exp.dTomato and Exp.PIAS4 (0.5–2 µg/µl) were prepared and mixed with Lipofectamine™ 2000 (ThermoFisher Scientific) (1–8 µl) in serum-free medium. After a 15-minute incubation at room temperature, 250 µl of the transfection mixture was added to each well and incubated for 48 h. Cells were then assessed by fluorescence microscopy for transfection efficiency and cells expressing plasmids are selected using 2 µg/µl puromycin for further couple of passages to establish stable cell lines.

### Western blot analysis

Proteins from naked mole rat intestines (ileum and colon), MCF10 and MCF7 cells were extracted using RIPA lysis buffer with protease inhibitors and quantified by BCA assay (ThermoFisher Scientific). 10% SDS-PAGE separated equal amounts of protein (40 µg), transferred to PVDF membranes (Merck, UK), and confirmed by Ponceau S staining. Membranes were blocked with 5% BSA for 1 h, then incubated overnight at 4 °C with primary antibodies: Anti β-actin (Mouse monoclonal, 1:5000; Sigma Aldrich, UK), α-Tubulin (Rabbit polyclonal, 1:1000; Cell Signalling Technology, UK), Anti PIAS4 (Rabbit polyclonal, 1:500/1000; Invitrogen, UK), Anti Bcl-2 (Rabbit polyclonal, 1:1000; Cell Signalling Technology, UK). After washing, membranes were incubated with IRDye-conjugated secondary antibodies and visualised using the LI-COR Odyssey System. Band intensities were quantified using LI-COR Empiria Studio v2.3 software.

### MTT assay

MTT assay (3-(4,5-dimethylthiazol-2-yl)−2,5-diphenyltetrazolium bromide) was used to determine the IC₅₀ of DOX using MCF7 cells expressing Exp.dTomato and Exp.PIAS4. Cells (1 × 10⁴ml) were seeded in 96-well plates, incubated for 24 h, and treated with DOX (100 µM–30 nM) for 48 h. After drug exposure, MTT solution was added and incubated for 4 h. Formazan crystals were dissolved in DMSO, and absorbance at 540 nm was measured using a Multiskan plate reader. IC₅₀ values were calculated with GraphPad Prism.

### Colony formation

StableMCF7 cells expressing Exp.dTomato and Exp.PIAS4 (5 × 10² cells/well) were seeded in triplicate into 6-well plates and incubated for 24 h at 37 °C to allow adherence. Cells were then treated with 1 µM doxorubicin (DOX) for 48 h, followed by incubation in fresh growth medium for 10 days at 37 °C in 5% CO₂ without media change. Colonies were fixed with 95% methanol: glacial acetic acid (7:1) for 10 min, stained with 0.5% crystal violet for 10 min at room temperature, washed, and air-dried overnight. Colonies were visualised using an Olympus CKX53 inverted microscope at 10× objective, and representative images were captured. Stained colonies were quantified using ImageJ software (NIH, USA) to determine colony area and intensity. Colony numbers were measured and quantified from three replicates to ensure reproducibility.

### Cell invasion assay

The cell invasion assay was performed to assess the invasive potential of treated and untreated MCF7 cells (Exp. dTomato and PIAS4 transfected) following DOX exposure. Briefly, cells were serum-starved for 24 h before the assay, and 1.0 × 10⁶ cells/ml were prepared in serum-free medium. All plates and reagents were equilibrated to 25 °C before use. Cell invasion was assessed according to the manufacturer’s protocol using the QCM™ ECMatrix™ Cell Invasion Assay (fluorometric format; Merck-Millipore, UK). Fluorescence was measured using a Multiskan plate reader (ThermoFisher Scientific) at a wavelength of 480/520 nm. Background fluorescence from blank wells was subtracted, and results were expressed as relative fluorescence units (RFU). Each condition was assayed in technical triplicate and three independent experiments to ensure reproducibility.

### Naked mole rats

The naked mole rats were bred at the University of Bradford in a purpose-built research facility with licensed authority to bred, supply and use animals under the Animals (Scientific Procedures) Act 1986 complying with national Home Office guidelines for use animals in this study. They have customised, linked cages which attempt to replicate their natural colony housing arrangements and are kept in family groups with one breeding queen. The room conditions are ~ 30 °C with relative humidity of ~ 55%, red lighting is used during the day to enable husbandry procedures whilst protecting their eyes from light exposure. Fresh food is provided every few days and consists of fruit and veg, with seeds, nuts, and popcorn for variety, and a nutritional mash diet provided for the pregnant queen and young offspring. Euthanasia involves anaesthetising the animal and dislocating its neck, death is confirmed by exsanguination. Schedule 1 work is covered under the establishment licence. Intestinal samples (ileum and colon) were stored in RNA*later*™ (ThermoFisher Scientific) stabilisation solution after dissection and cleaning. For protein analysis the same tissue samples were stored at −80 °C after dissection and cleaning.

### Statistical analysis

All experiments were performed in three independent biological replicates. Data are presented as mean ± standard deviation (SD). Statistical significance between two groups was assessed using an unpaired two-tailed Student’s t-test, while comparisons among multiple groups were performed using a one-way ANOVA test. The half-maximal inhibitory concentration (IC₅₀) values for DOX treatment were calculated using nonlinear regression analysis in GraphPad Prism 10 (GraphPad Software, USA). P-values < 0.05 were considered statistically significant. Graphs and statistical analyses were generated using GraphPad Prism.

## Results

### SUMOylation pathway gene expression analysis in NMR tissue and human breast cell lines

Sequence alignment between Heterocephalus glaber (NMR) and Homo sapiens (Human) was performed for SUMOylation machinery genes. Database analysis was performed using NCBI’s Conserved Domain Database (CDD) to identify structural motifs within SUMO-related genes and proteins (SUMO1, SUMO2, SUMO3, SAE1, UBA2, SENP isoforms, and PIAS isoforms) in NMR and human. Sequence identity between human and naked mole-rat SUMO genes ranged from 82.57% to 95.14% (Supplementary Table 2), and the protein ranged from 81.56% to 100% alignment (Supplementary Table 3), indicating a highly conserved interface across species The resulting summary table presents gene and protein names, total scores, E-values, identity percentages, and accession numbers corresponding to homologous subfamily architectures (Supplementary Tables 2 and 3).

**Fig. 1:**
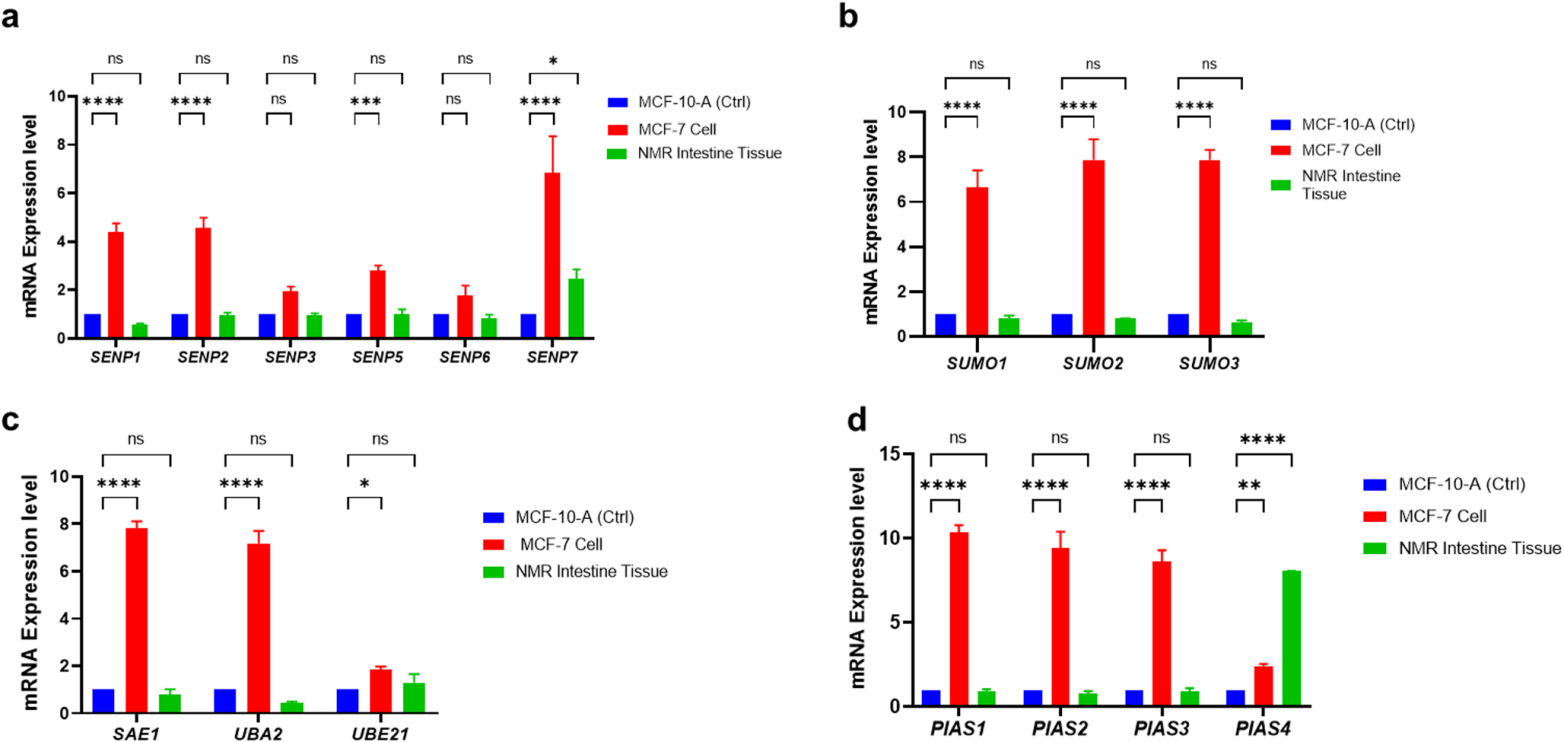
SUMOylation genes expression levels in human breast cells and naked mole rat intestinal tissues. Gene expression in MCF-10-A (control breast), MCF-7 cell lines, and NMR intestinal tissue. **(a)** SENP isoforms **(b)** SUMO1, SUMO2, and SUMO3 **(c)** SAE1, UBA2, and UBE21 **(d)** PIAS isoforms gene expression. qPCR was used to confirm SUMOylation gene expression levels. Mean and Error bars correspond to SD from three repeats of individual experiments (*n* = 3). Statistical analysis was performed by ordinary two-way ANOVA and indicated a significant difference in where *indicates *p* < 0.05, **indicates *p* < 0.01, ***indicates *p* < 0.001, and ****indicates *p* < 0.0001

The expression of SUMOylation genes was evaluated in NMR intestinal tissue, human breast cancer (MCF-7) and normal breast (MCF-10-A) cell lines by qPCR. The results revealed that SUMOylation machinery (*SENP1–7*,* SUMO1/2/3*,* SAE1*,* UBA2*,* UBE21*,* PIAS1-3*) was relatively low in NMR tissues and MCF-10 A (Fig. 1a, b, c, d). In contrast, the SUMOylation pathway component, PIAS4 gene, is significantly higher in NMR intestinal tissues compared to both MCF-10 A and MCF-7 cells (Fig. 1d).

### Effect of PIAS4 overexpression and DOX treatment on Bcl-2 expression

**Fig. 2:**
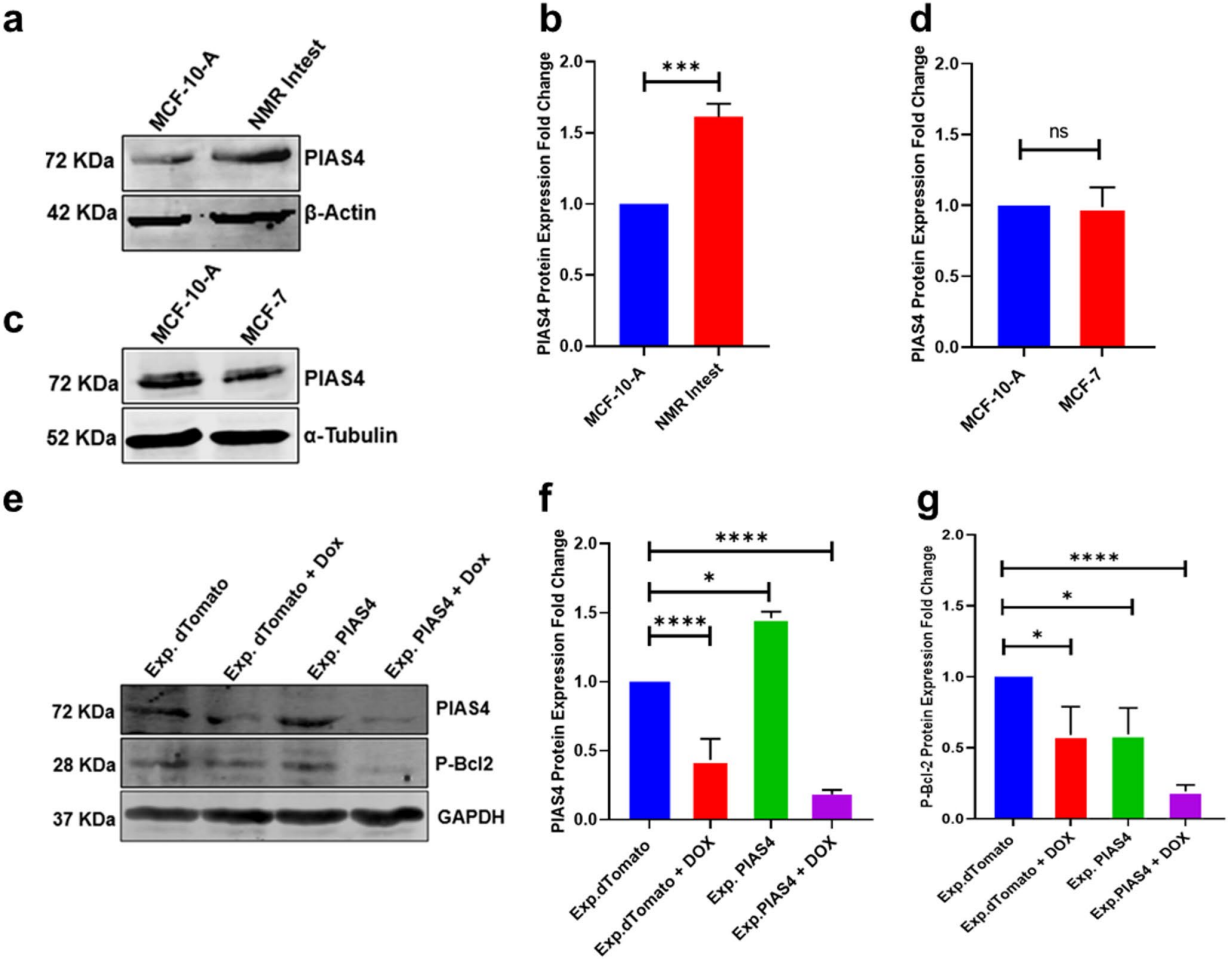
DOX treatment and PIAS4 overexpression affects protein expression levels in MCF-7 cells. **(a)** PIAS4 protein expression in MCF-10-A cells and NMR intestinal tissue, **(b)** Histogram analysis of (a). **(c)** PIAS4 protein expression in MCF-10-A and MCF-7 cells. **(d)** Histogram analysis of (**c**). **(e)** PIAS4 and Bcl2 protein expression levels in MCF-7 cells stably expressing Exp.dTomato (control) or Exp.PIAS4, with or without DOX treatment. (**f**) Histogram of PIAS4 expression levels from (**e**). **(g)** Histogram of Bcl-2 protein levels from (**e**). All Western blots were derived from the same lysate, performed concurrently, and represent three independent experiments (*n* = 3). Protein expression levels were normalised either α-Tubulin, GAPDH or β-actin. Data are presented as mean ± standard deviation (SD) from three independent experimental repeats. Statistical analysis was performed using one-way ANOVA. *p < 0.05, ***p < 0.001, ****p < 0.0001 indicate statistically significant differences

Western blot analysis revealed significant increase in PIAS4 protein expression in NMR intestinal tissue compared to MCF-10-A control cells (Fig. 2a, b) corroborating qPCR analysis. However, there was no change in PIAS4 protein expression in MCF-7 cancer cells relative to the MCF-10-A control cells (Fig. 2c, d). While PIAS4 mRNA levels were significantly higher in MCF7 cells compared to MCF10A in Fig. 1d, the corresponding protein expression did not show this difference. This variation may reflect regulatory control at the post-transcriptional level, impacting PIAS4 translation and/or stability. Maybe post-translational modifications may change epitope accessibility, potentially impacting antibody-based detection such as Western blotting. MCF-7 cells stably expressing either control expression plasmid (Exp.dTomato) or PIAS4 (Exp.PIAS4) revealed reduced Bcl-2 expression. Furthermore, upon treatment with DOX significant reduction in expression of both PIAS4 and Bcl-2 proteins was observed (Fig. 2e, f, g). This data also suggest that treatment of MCF-7 cells with DOX is affecting the stability of the PIAS4 protein expression levels or there might be change in post-translational modification state which is affecting the detection of the PIAS4 protein.

### Effects of PIAS4 overexpression on MCF-7 cell proliferation

**Fig. 3:**
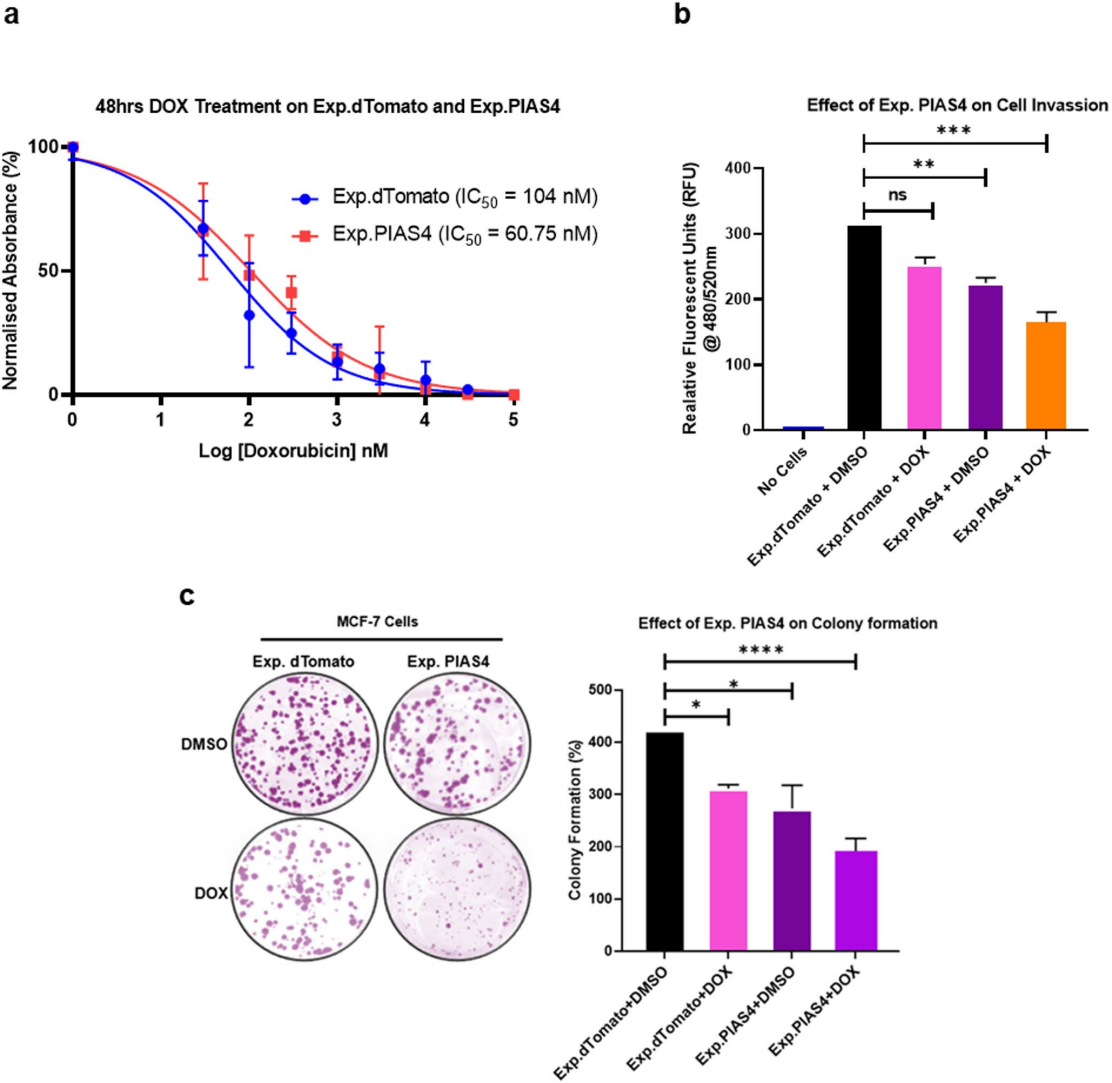
Functional effects of PIAS4 overexpression in MCF-7 cells with and without DOX treatment. **(a)** IC₅₀ of 48-hour DOX treatment in MCF-7 cells transfected with Exp.dTomato or Exp.PIAS4. MCF-7 cells stably expressing Exp.dTomato or Exp.PIAS4 plasmids following 48-hour DOX treatment **(b)** Cell invasion was assessed using fluorescence measurements and reported as relative fluorescence units (RFU) at 480/520 nm. **(c)** Colony formation images were analysed using ImageJ^®^ software for quantification. Data are presented as mean ± SD from three independent experiments (*n* = 3). Statistical analysis was performed in GraphPad Prism using one-way ANOVA. *p < 0.05, **p < 0.01, ***p < 0.001, ****p < 0.0001

MCF-7 cells overexpressing PIAS4 (Exp.PIAS4) showed a significant reduction in the DOX IC₅₀ (60.75 nM) compared to MCF-7 cells overexpressing dTomato (Exp.dTomato) (104 nM) (Fig. 3a). MCF-7 cells stably expressing PIAS4 (Exp.PIAS4) reveal decreased invasion and colony formation with or without DOX treatment, compared to expression control (dTomato) stably expressing MCF-7 cells. These results indicate increased sensitivity to DOX treatment when PIAS4 is expressed (Fig. 3b, c).

## Discussion

The current study introduces perspectives on the function of the SUMO E3 ligase PIAS4 in the development and resistance to chemotherapy of breast cancer. The findings of our study demonstrate that *PIAS4* gene expression is higher in NMR intestinal tissue, and when it is recapitulated (overexpressed) in MCF-7 breast cancer cells, it reduces the colony formation and cell invasion. Moreover, the study suggests that DOX treatment and overexpression PIAS4 reduces Bcl-2, an anti-apoptotic protein. These results are consistent with mounting evidence that SUMOylation, a reversible post-translational modification, is essential for plasticity and resistance to the treatment of cancer cells. As E3 ligases, PIAS proteins in particular, PIAS1 and PIAS4, help SUMO conjugate to a range of substrates, including transcription factors and DNA repair proteins [7, 8].

PIAS4 has been involved in regulating *p53* signalling, DNA double-strand break repair via interaction with *BRCA1*, and the modulation of inflammatory pathways through NF-κB inhibition [15]– [16]. These functions are particularly relevant in cancer, where dysregulated DNA repair and evasion of apoptosis are key hallmarks of disease progression and therapeutic failure.

Our findings suggest that PIAS4 overexpression reduced the IC₅₀ of DOX in MCF-7 cells, suggesting that PIAS4 may be mediating cellular stress or possibly regulating DNA repair pathways [17]. PIAS4 overexpression markedly reduced colony formation and invasion, supporting its tumour-suppressive potential. Interestingly, this was accompanied by a decrease in Bcl-2 expression, suggesting activation of pro-apototic mechanisms. Through SUMOylation, PIAS4 may influence transcription factors such as p53 or NF-κB, altering the balance between pro- and anti-apoptotic gene expression. Bcl-2 overexpression has been previously associated with poor prognosis and resistance to chemotherapy in breast cancer [18, 19]. PIAS4 may regulate Bcl-2 transcriptionally or via SUMOylation-dependent modulation of upstream regulatory proteins, although this requires further mechanistic clarification.

Interestingly, NMR is known for cancer resistance and genomic stability and showed relatively high levels of PIAS4 in intestinal tissues, despite low expression levels of other SUMO pathway components. This suggests a possible context-dependent role of PIAS4 in different species. From a translational perspective, the current study suggests that PIAS4 could serve as both a biomarker and a therapeutic target in breast cancer. Pharmacological modulation of SUMOylation has shown promise in preclinical cancer models, breast cancer pathways and specific targeting of PIAS4 could sensitise tumours to DNA-damaging agents such as DOX [10, 20, 21]. As suggested by their nomenclature PIAS’s functions as a suppressor of the STAT mediated pathway, which are important regulators of critical cellular processes such as proliferation, differentiation, and apoptosis. However, due to the pleiotropic functions of SUMOylation, systemic inhibition poses the risk of toxicity. Therefore, selective PIAS4 modulators may offer more precise strategies for therapeutic intervention.

Finally, while the findings provide a strong rationale for further studies investigating PIAS4, this study is limited by its in vitro design including limited analysis of signalling pathways such as apoptosis and should be expanded to other breast cancer cell lines. In vivo validation in breast cancer xenograft or patient-derived organoid models will be essential to confirm the clinical relevance of PIAS4-mediated effects. Additionally, proteomic analyses could help identify PIAS4 substrates and downstream effectors, enhancing our understanding of its molecular network in breast cancer.

## Conclusion

**Fig. 4:**
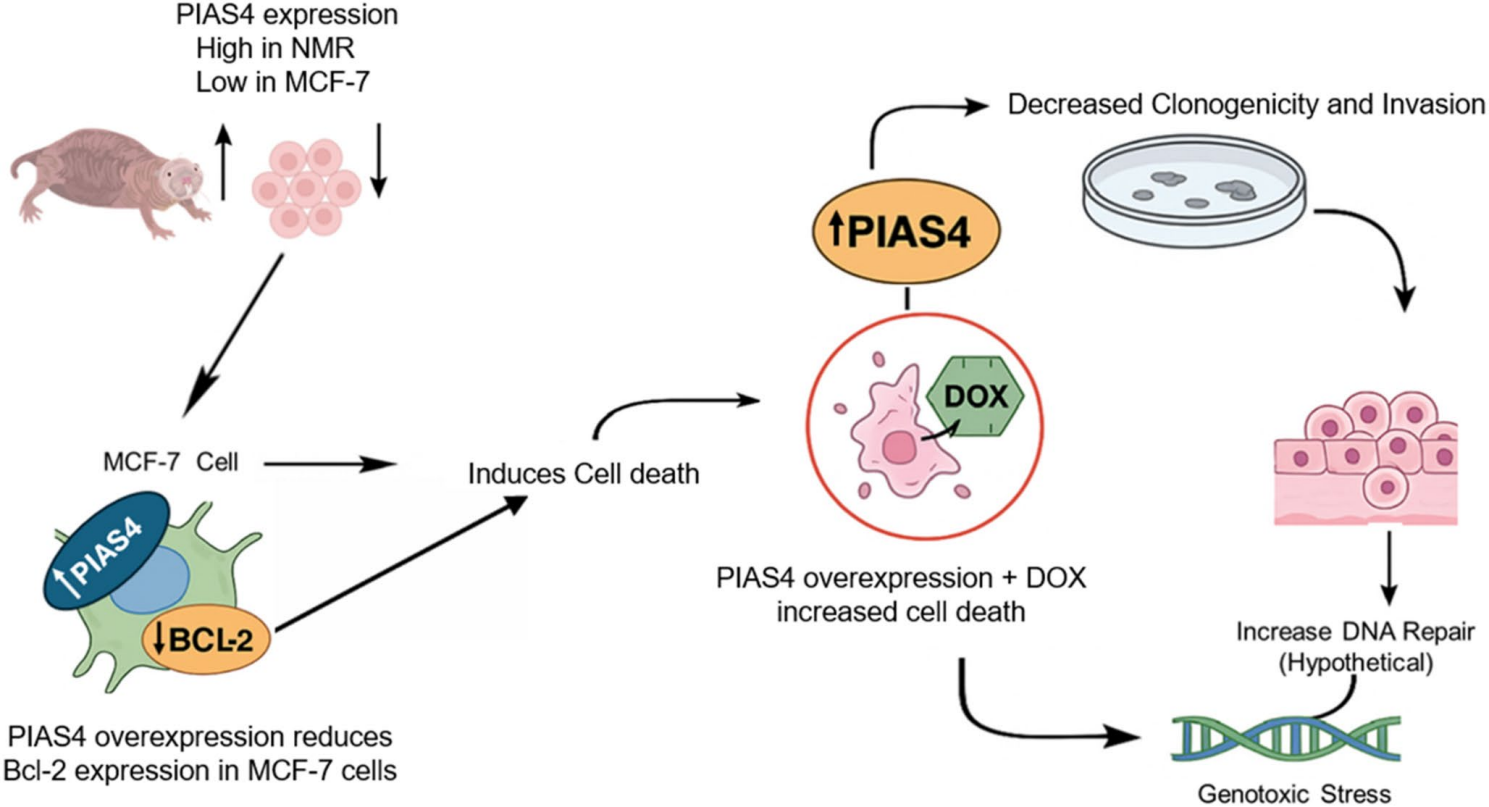
Schematic diagram for the PIAS4-mediated mechanisms in MCF-7 breast cancer cells. The increased level of PIAS4 in the naked mole-rat intestine, while low expression in MCF-7 cells. Recapitulated PIAS4 NMR levels in MCF-7 human breast cancer cells by overexpressing after which it downregulates anti-apoptotic protein Bcl-2. Exposure to doxorubicin (DOX)-induced cell death which is further enhanced by PIAS4 expression. PIAS4 overexpression caused a in decrease colony formation and cell invasion. PIAS4 may enhance DNA repair mechanisms, supporting cellular adaptation under genotoxic stress [16]

This study demonstrates a role of PIAS4 in human breast cancer cells, where increased expression decreases invasiveness, colony formation and doxorubicin IC_50_. Higher expression of PIAS4 was found in the NMR intestinal tissue compared to other SUMOylation genes, suggesting a potential evolutionary adaptation linked to genome maintenance. These findings also suggest that PIAS4 regulates expression of anti-apoptotic protein Bcl-2. Considering the significant role in cancer development and progression, PIAS4 may be identified as a promising biomarker and a potential therapeutic target specifically within the context of breast cancer (summarised in Fig. 4). Consequently, the study indicates that further research is required for clarification regarding the mechanism of PIAS4 action, which can be a potential strategy to enhance cancer treatments and/or reduce drug resistance.

## Supplementary Information

Below is the link to the electronic supplementary material.

Supplementary Material 1

## Data Availability

No datasets were generated or analysed during the current study.
