# Supplementary material for: SUMOylation machinery protein, PIAS4 role in breast cancer cell proliferation and drug sensitivity
Source: Mol Biol Rep. 2026 Jan 30;53(1):336. doi: 10.1007/s11033-025-11423-0 (PMC12858598; doi:10.1007/s11033-025-11423-0)
Supplement: Supplementary file 1 — Supplementary Material 1 [file 11033_2025_11423_MOESM1_ESM.docx]

Supplementary information

Supplementary Table 1: Describs the list of the forward (F) and reverse (R) primer sequences used for amplification of genes involved in the SUMOylation pathway, including *SUMO* isoforms (*SUMO1–3*), E1-activating enzymes (*SAE1, UBA2*), E2-conjugating enzyme (*UBE2I*), SUMO-specific proteases (*SENP1–7*), and SUMO E3 ligases (*PIAS1–4*). Primers were designed using NCBI Primer-BLAST and PrimerQuest™ and synthesised by Sigma-Aldrich (UK). The housekeeping gene B2M was included for normalisation of gene expression in qPCR analysis.

| *NMR (Heterocephalus glaber) Primers* | | |  |
| --- | --- | --- | --- |
| Gene | **Sequence (5’–3’)** | **Supplier** |  |
| *B2M* | **F:** GCCAGAGACTCCAAAGATTCA  **R:** GTTGGTATGCACCAGGAGATAG | Sigma-Aldrich, UK |  |
| *SUMO1* | **F:** GACACTGAGACGGGATTGTAAA  **R:** CATCTCCCACTGCAAGTCATAG | Sigma-Aldrich, UK |  |
| *SUMO2* | **F:** GCCCAAGGAAGGAGTCAAG  **R:** TGTGCAGGTGTGTCTGTTT | Sigma-Aldrich, UK |  |
| *SUMO3* | **F:** CGGCCTGTCGTAAGCATATAG  **R:** GAAAGCAGTGGAGCACAAATC | Sigma-Aldrich, UK |  |
| *SAE1* | **F:** TGAACTGGAGCAGTGAGAAAG  **R:** GCC AGGACAGAGATGTCATAAG | Sigma-Aldrich, UK |  |
| *UBA2* | **F:** AGTGATTGCCGGGTTGATAG  **R:** GCTTCTGTCTCTCCTTCTTCTG | Sigma-Aldrich, UK |  |
| *UBE2I* | **F:** CAGTGAGACTGCAGACCATAAA  **F:** GGAACTGGGTCTCAGGAATAAG | Sigma-Aldrich, UK |  |
| *SENP1* | **F:** TTCCGGTTCGGACTTTGTATC  **R:** GTTGAGCTGGGTATTGGAGTAG | Sigma-Aldrich, UK |  |
| *SENP2* | **F:** CTGCTTGGGCTTGGTATGTA  **R:** CGGCCACTTGGTCTTCTATT | Sigma-Aldrich, UK |  |
| *SENP3* | **F:** GAGGAAGAGGAGGAGGATGAA  **R:** CTATAAGCGGCATGTGCTGA | Sigma-Aldrich, UK |  |
| *SENP5* | **F:**CAAGAGCCTGACAGATGCTATAC  **R:** GATTAGGAGTGATTGAGGCGTC | Sigma-Aldrich, UK |  |
| *SENP6* | **F:** AGTGATTGTGGCGTGTATGT  **R:**CATAAGGACACTTCCTCCACAG | Sigma-Aldrich, UK |  |
| *SENP7* | **F:** CCCAGTACGGCTTGAAGAAA  **R:**TCTTATCCATTGTCGTGGGTTC | Sigma-Aldrich, UK |  |
| *PIAS1* | **F:** GGTCTGTCCTGTCTGTGATAAG  **R:** GAGGAGGGAGGTGTTGTAATG | Sigma-Aldrich, UK |  |
| *PIAS2* | **F:** CGGTGAGGTAAGCAAGAAGAA  **R:**CCCAGTAGGACAGCCATAATAAA | Sigma-Aldrich, UK |  |
| *PIAS3* | **F:** CTCGCATCCACTTCTAGTCATC  **R:** AGCGGTCAGTTTCTCCTTTATC | Sigma-Aldrich, UK |  |
| *PIAS4* | **F:** GATGAACGAGAAGAAGCCTACC  **R:** CCTCTTCCTCATCCTCCTCTT | Sigma-Aldrich, UK |  |
| *Human (Homo sapiens) Primers* | | |  |
| Gene | | **Sequence (5’–3’)** | **Supplier** |
| *B2M* | **F**: TCGCGCTACTCTCTCTTTCT  **R**: CGGCAGGCATACTCATCTTT | Sigma-Aldrich, UK |  |
| *SUMO1* | **F**: CAAAAGAACTGGGAATGGAG  **R**: CAGATGTTTCAAAGAGATGGG | Sigma-Aldrich,UK |  |
| *SUMO2* | **F**: GTTAGGAAATGGAGGATGAAG  **R**: CAGAGTTCTGGAGTAAAGAAG | Sigma-Aldrich, UK |  |
| *SUMO3* | **F**: CAATGACCCATCATCTCTTG  **R**: TGAATAAACACAACAGAGCC | Sigma-Aldrich, UK |  |
| *SAE1* | **F**: GATACACATTTGCCAATCTAGG  **R**: AGAAGACCACCTTCTTTTTG | Sigma-Aldrich, UK |  |
| *UBA2* | **F**: CTGAAGTACAAAGTCAAGGAG  **R**: CTTTACATCTAGAACCTGCTG | Sigma-Aldrich,UK |  |
| *UBE2I* | **F**: CCGCTCCCAGAATGTCTTATT  **R**: AAGGTTCACGTCCTTCGTTAG | Sigma-Aldrich, UK |  |
| *SENP1* | **F**: GATTTTATCTTCCAGGCAAGG  **R**: GCTTGGATTATAAGCTGCAC | Sigma-Aldrich, UK |  |
| *SENP2* | **F**: GCAAAGAGAGGGACAGAAGAA  **R**: CCGATGAATAGGCACCAGAATA | Sigma-Aldrich, UK |  |
| *SENP3* | **F**: GCAGAGGCGGTAAAGAAAGA  **R**: GGAAGAGGCAAGAGAGGAAAG | Sigma-Aldrich, UK |  |
| *SENP5* | **F**: CCACCACTCCCAGCTAATTT  **R**: CAGAGCAGGTCATCAGTGTATC | Sigma-Aldrich, UK |  |
| *SENP6* | **F**: ATCAGCCTACTCCTCCTCTATC  **R**: GTCTGGTTCGTCTAGCTGTATC | Sigma-Aldrich, UK |  |
| *SENP7* | **F**: CGGTTGCTACTCCCTTTCTATT  **R**: TGTGACGAGTCCATGTTCTTAC | Sigma-Aldrich, UK |  |
| *PIAS1* | **F**: ACCCAATCTTTGTGTGAAAG  **R**: TTCTTCCAATTTCTGCAGTC | Sigma-Aldrich, UK |  |
| *PIAS2* | **F**: GTACTGTCCTCCTATGTTTTTG  **R**: AAGTGAGTCCTCCTTTAGTC | Sigma-Aldrich, UK |  |
| *PIAS3* | **F**: GATCAAGGAGAAATTGACTGC  **R**: GGCTTCTTCTCATTCATCTG | Sigma-Aldrich, UK |  |
| *PIAS4* | **F**: GAGAATCTGTTACTCAGACAC  **R**: GCTTATTGGAGGGGTAGTAG | Sigma-Aldrich, UK |  |

**Supplementary Table 2:** Summary of SUMOylation genes pairwise alignment between Homo sapiens and Heterocephalus glaber.

| Gene | Score | Error | Identities % | ACC.Len | Accession |
| --- | --- | --- | --- | --- | --- |
| *SUMO1* | 1374 | 0.0 | 92.55 | 1245 | Query_65267 |
| *SUMO2* | 549 | 6e-160 | 95.14 | 521 | Query_42207 |
| *SUMO3* | 886 | 5e-143 | 90.72 | 1626 | Query_19995 |
| *UBE2I* | 1571 | 0.0 | 87.52 | 2666 | Query_265295 |
| *SAE1* | 1304 | 0.0 | 89.82 | 1837 | Query_53017 |
| *UBA2* | 2969 | 0.0 | 90.20 | 2545 | Query_299945 |
| *SENP1* | 1914 | 0.0 | 92.23 | 4659 | Query_43745 |
| *SENP2* | 2973 | 0.0 | 87.17 | 3047 | Query_228665 |
| *SENP3* | 2924 | 0.0 | 90.82 | 3134 | Query_17463 |
| *SENP5* | 3037 | 0.0 | 82.57 | 5623 | Query_13795 |
| *SENP6* | 4993 | 0.0 | 87.57 | 4409 | Query_4185 |
| *SENP7* | 4523 | 0.0 | 86.56 | 5037 | Query_1451 |
| *PIAS1* | 4017 | 0.0 | 89.25 | 6342 | Query_12561 |
| *PIAS2* | 2816 | 0.0 | 95.07 | 11415 | Query_35353 |
| *PIAS3* | 3450 | 0.0 | 88.34 | 2902 | Query_37251 |
| *PIAS4* | 1895 | 0.0 | 88.86 | 3069 | Query_339167 |

**Supplementary Table 3:** Summary of SUMOylation proteins pairwise alignment between NMR and Humans.

| Protein | Score | Error | Identities% | ACC.Len | Accession |
| --- | --- | --- | --- | --- | --- |
| SUMO1 | 211 | 3e-77 | 100.00 | 154 | Query-62295 |
| SUMO2 | 161 | 2e-58 | 92.57 | 108 | Query_54979 |
| SUMO3 | 174 | 7e-63 | 96.91 | 108 | Query_23203 |
| UBE2I | 331 | 1e-123 | 100.00 | 158 | Query_52531 |
| SAE1 | 487 | 1e-180 | 91.63 | 285 | Query_52535 |
| UBA2 | 1288 | 0.0 | 96.88 | 640 | Query_18977 |
| SENP1 | 1198 | 0.0 | 92.39 | 717 | Query_19691 |
| SENP2 | 1094 | 0.0 | 89.47 | 589 | Query_11299 |
| SENP3 | 1145 | 0.0 | 97.21 | 572 | Query_37089 |
| SENP5 | 899 | 0.0 | 81.56 | 750 | Query_40673 |
| SENP6 | 1842 | 0.0 | 84.64 | 1150 | Query_42727 |
| SENP7 | 1743 | 0.0 | 83.49 | 1046 | Query_53401 |
| PIAS1 | 1310 | 0.0 | 98.45 | 651 | Query_30977 |
| PIAS2 | 1127 | 0.0 | 98.05 | 622 | Query_40887 |
| PIAS3 | 1214 | 0.0 | 97.09 | 616 | Query_43921 |
| PIAS4 | 961 | 0.0 | 87.34 | 553 | Query_22351 |


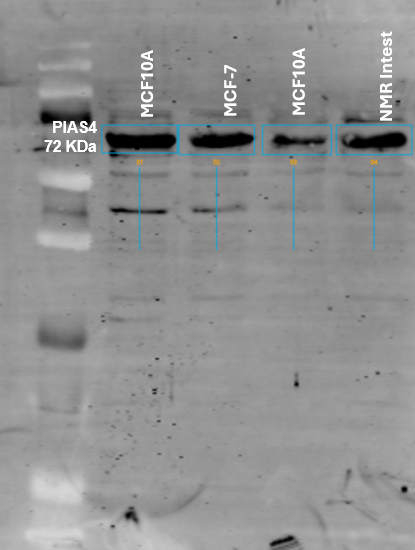


**Supplementary Figure 1:** PIAS4 protein expression in MCF-10-A, MCF-7 cells and NMR Intestinal Tissue.


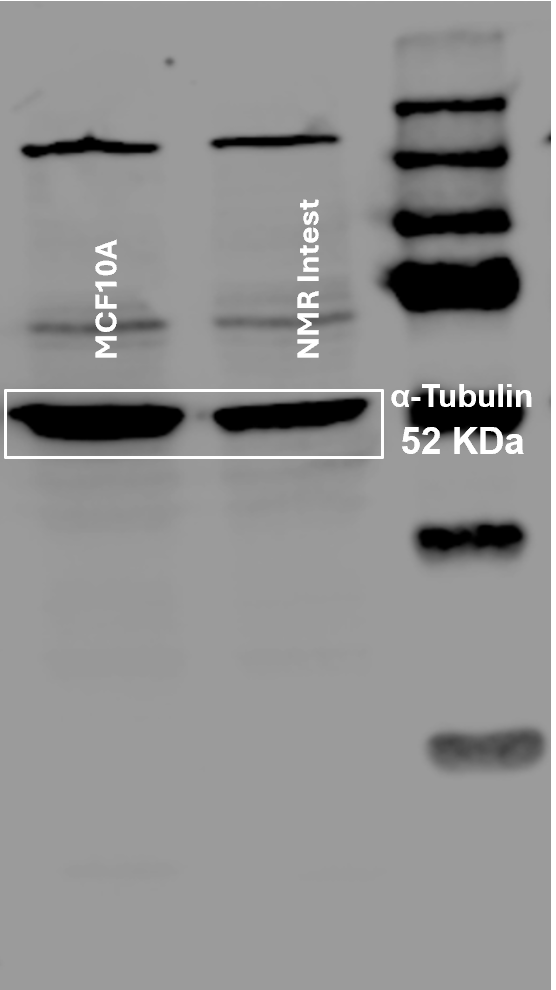


**Supplementary Figure 2:** α-Tubulin protein expression levels in MCF-10-A and NMR Intestinal tissue. An α-Tubulin blot used to normalise to PIAS4 protein expression levels in MCF-10-A cell and NMR Intestinal Tissue in **Supplementary figure 1** .


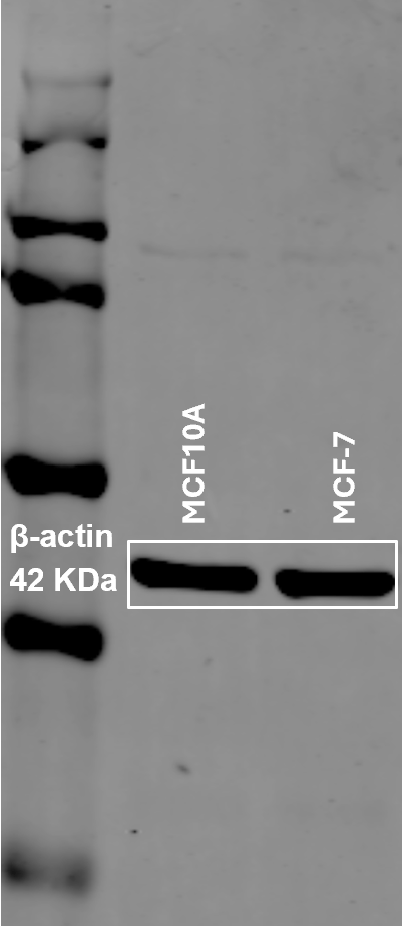


**Supplementary Figure 3:** β-actin protein expression levels in MCF-10-A and MCF-7 cells. An β-actin blot used to normalise to PIAS4 protein expression levels in MCF-10-A, and MCF-7 cells in **Supplementary figure 1** .


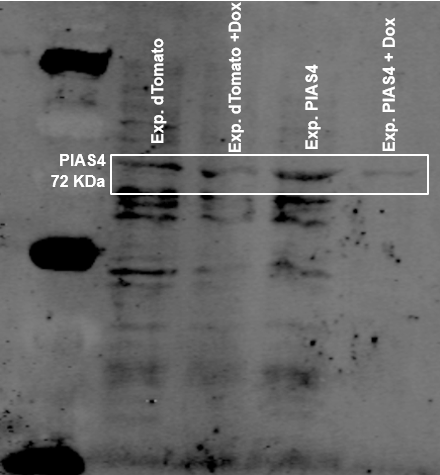


**Supplementary Figure 4:** PIAS4 protein expression levels in MCF-7 cells transfected with Exp.dTomato (control) or Exp.PIAS4, with or without DOX treatment.


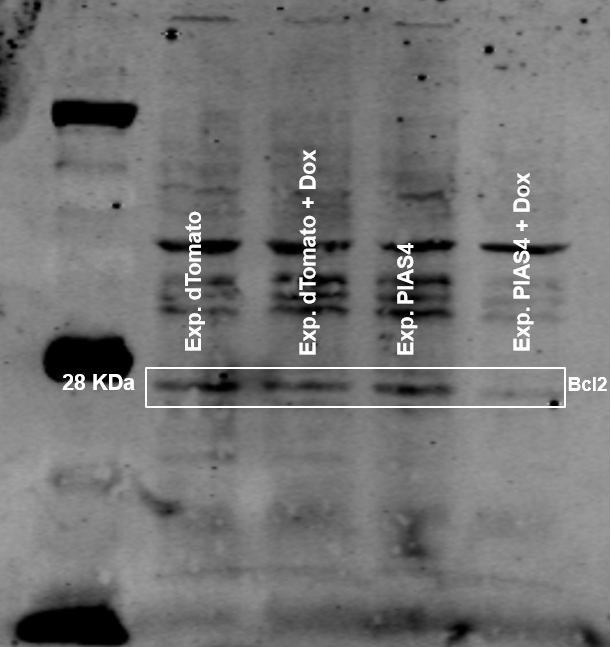


**Supplementary Figure 5:** Bcl2 protein expression levels in MCF-7 cells transfected with Exp.dTomato (control) or Exp.PIAS4, with or without DOX treatment.


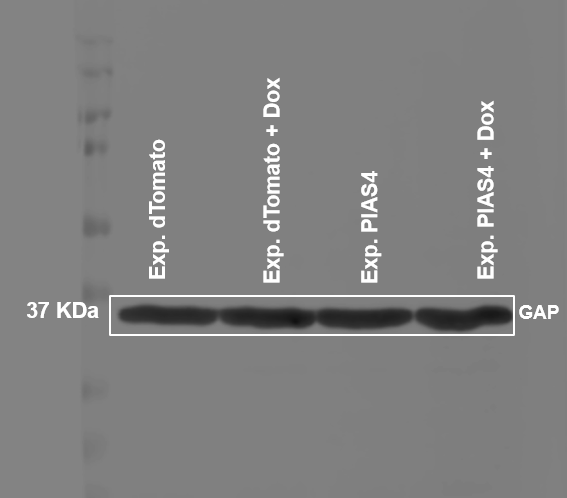


**Supplementary Figure 6:** GAPDH protein expression levels in MCF-7 cells transfected with Exp.dTomato (control) or Exp.PIAS4, with or without DOX treatment. GAPDH blot used to normalise to PIAS4 and Bcl2 protein expression levels in **Supplementary figures 4 and 5**.
